# Effects of a Short-Term Meal Replacement Hypocaloric Diet in Subjects with Obesity and High Fatty Liver Index

**DOI:** 10.3390/nu14245353

**Published:** 2022-12-16

**Authors:** Daniel de Luis, David Primo, Olatz Izaola, Juan Jose Lopez

**Affiliations:** Center of Investigation of Endocrinology and Nutrition, Department of Endocrinology and Investigation, Medicine School, Hospital Clinico Universitario, University of Valladolid, 47003 Valladolid, Spain

**Keywords:** fatty liver index, obesity, partial meal replacement diet, transaminases

## Abstract

Introduction: Dietary changes play a role in metabolic response of patients with metabolic-associated fatty liver disease, and there is little evidence on the use of partial meal replacement (pMR) diets in this pathology. Aim: We decided to evaluate the modifications in transaminases levels after a pMR hypocaloric diet in subjects with obesity and elevated fatty liver index (FLI). Material and methods: A sample of 606 patients with obesity and FLI ≥ 60 were enrolled and treated during 3 months with a pMR diet. Patients were divided as group I (Alanine amino transferase (ALT) normal) or group II (ALT ≥ 43 UI/L). Results: Body mass index, body weight, total fat mass, waist circumference, blood pressure, fasting glucose, total cholesterol, Low-density lipoprotein (LDL) cholesterol, triglycerides, insulin, Homeostasis Model assessment (HOMA-IR), and FLI index improved significantly in the total group with pMR diet, without differences between group I and II. ALT, aspartate aminotransferase activity (AST), Gama glutamine transferase (GGT), and ratios of AST/ALT improved in both groups, too. This improvement was higher in group II (deltas group I vs. deltas group II); ALT (−4.2 ± 0.9 UI/L vs. −32.1 ± 5.7 UI/L: *p* = 0.01), AST (−4.8 ± 1.8 UI/L vs. −14.1 ± 1.9 UI/L: *p* = 0.02), GGT (−4.8 ± 1.4 UI/L vs. −37.1 ± 4.2 UI/L: *p* = 0.01), and AST/ALT ratio (−0.04 ± 0.002 units vs. −0.19 ± 0.04 units: *p* = 0.01). Conclusions: We reported that a pMR diet is an effective method to lose weight and to improve metabolic parameters in patients with obesity and high FLI. The decrease in liver parameters was greater in patients with ALT ≥ 43 UI/L.

## 1. Introduction

The increasing incidence of obesity is related to many obesity-related health comorbidities, including diabetes mellitus type 2, cardiovascular events, blood hypertension, hyperlipidemia, and metabolic-associated fatty liver disease (MAFLD). MAFLD is defined as the storage of lipids, primarily in the form of triacylglycerol, in subjects who do not drink significant amounts of alcohol and in whom other known causes of steatosis, such as certain toxins and drugs, have been excluded [1].

Weight loss is considered the most useful treatment for MAFLD. Decrease in body weight by 5% is related with 25% steatosis improvement, >6–9% with reduction in steatohepatitis and >10% with regression of liver fibrosis [2]. The most common method used in the treatment of obesity is a low-calorie diet with exercise, with the goal of reaching a weight loss of at least 5% in a short-term period [3]. An option among low-calorie diets is the diets of partial meal replacements (pMRs). Recently, a meta-analysis has reported that pMRs diets produced superior weight loss than conventional diets, 7% vs. 3% in 3 months, compared to traditional energy-restricted food-based diets [4]. Despite the effectiveness of weight loss for the treatment of MAFLD, studies with pMRs diets are scarce and have diverse designs in this type of patients. For example, Deibert et al. [5] reported a comparable effect on liver fat by magnetic resonance spectroscopy with a pMR diet vs. a comprehensive lifestyle intervention. Baltry et al. [6] demonstrated a similar effect on the liver histology of a food-based diet vs. a pMR diet in subjects with severe obesity prior to bariatric surgery.

In clinical practice, it is important to use a noninvasive pathway to evaluate change in hepatic steatosis during weight loss treatment. For example, the fatty liver index (FLI) is an emerging algorithm that has been created for the diagnosis of fatty liver in the general population [7]. Four variables are involved in FLI: body mass index (BMI), waist circumference, gamma glutamyl transpeptidase (GGT), and serum triglyceride (Tg) levels. This index achieved an accuracy of 0.84 in the detection of fatty liver, considering FLI values < 30 not indicative of MAFLD (sensitivity of 87% and negative likelihood ratio = 0.2), FLI values ≥ 60 indicative of NAFLD (specificity of 86%), and positive likelihood ratio = 4.3) [7]. Serum aspartate aminotransferase (AST) and alanine aminotransferase (ALT) are useful for monitoring subjects with MAFLD. The influence of treatments in liver status might be evaluated with these biomarkers, in order to facilitate follow-up with an easily accessible test via venipuncture [1]. In general, the decrease in its levels means a decrease in the fat content of the liver, in the context of patients with MAFLD [2].

Taking to account the evidence that dietary changes play a main role in the metabolic response of subjects with MAFLD and the little evidence on the use of pMR diet in this pathology, we decide to evaluate the modifications in transaminases levels after a pMR diet in subjects with obesity and elevated FLI.

## 2. Subjects and Methods

### 2.1. Subjects and Clinical Investigation

We recruited 606 Caucasian subjects with obesity. In this design, we prescribed a pMR diet with a normocaloric hyperproteic formula to subjects with obesity (Table 1) for 3 months. All participants agreed to participate in the trial, and all these subjects signed an informed consent form. The inclusion criteria for this design were the following: obesity assessed as body mass index ≥30 kg/m^2^ and a fatty liver index (FLI) ≥60 units. Subjects with obesity with one or more of the following data were excluded: a severe illness (e.g., chronic kidney disease, heart failure, previous cardiovascular events, malignant tumours, hepatitis B, C, cytomegalovirus, Epstein–Barr infections, nonorgan-specific autoantibodies and hereditary defects (iron and copper storage diseases and alpha 1-antitrypsin deficiency)), and history of active alcoholism. Following a caloric restriction in the previous 6 months or active treatments with statins, fibrates, and drugs against diabetes mellitus that modify insulin resistance were exclusion criteria, too.

The next adiposity parameters were registered at initial and at 3 months after dietary intervention (body weight, height, BMI, waist circumference, and fat mass by electrical bioimpedance). Blood pressure was determined, too. Both times, fasting blood samples were collected into ethylenediaminetetraacetic acid (EDTA)-coated tubes for analysis of alanine amino transferase (ALT), aspartate aminotransferase activity (AST), bilirubin and Gama glutamine transferase (GGT), basal fasting glucose, insulin, insulin resistance estimated by homeostasis-model- assessment for insulin resistance (HOMA-IR), total cholesterol, Low density lipoprotein cholesterol (LDL)-cholesterol, HDL-cholesterol, and plasma triglycerides.

### 2.2. Dietary Intervention

All obese subjects received the same nutritional instructions to follow a meal-replacement hypocaloric diet (pMR). This pMR diet was distributed in 6 daily meals: breakfast, lunch, dinner, and three snacks (breakfast morning snack, afternoon snack, after dinner snack). The lunch and dinner meals were substituted by a normocaloric hyperproteic formula (VEGESTART Complete^®®^, Vegenat, Badajoz, Spain) (Table 1), and the remaining servings were realized with normal foods. At basal time and after 3 months, all patients reported their dietary intakes at 72 h, in order to estimate the energy and macronutrients intakes. The macronutrients and calorie intakes were evaluated with nutritional software (Dietsource ^®®^, Nestlé, Geneva, Switzerland). Physical activity was self-evaluated with a questionnaire by each subject, and during the protocol, the allowed physical activity for patients was the following: aerobic physical exercise at least 3 or 4 times per week (60 min each); the proposed physical activities were walking, running, and cycling.

### 2.3. Biochemical Parameters

Fasting (12 h) venous blood samples (10 mL) were obtained from all participants by venepuncture. The samples were centrifuged, and the serum was used to carry out the determinations, and we froze all the samples at −80 °C until the biochemical determinations were carried out. Biochemical measurements, including transaminases, bilirubin, glucose, insulin, and lipid profile (LDL cholesterol, HDL-cholesterol, and triglyceride levels) using the COBAS INTEGRA 400 analyser (Roche Diagnostic, Basel, Switzerland). LDL cholesterol was determined using Friedewald formula (LDL cholesterol = total cholesterol-HDL cholesterol-triglycerides/5) [8]. HOMA-IR was obtained using these values (glucose × insulin/22.5) [9].

### 2.4. Adiposity Parameters, Arterial Blood Pressure and Fatty Liver Index

Central adiposity (waist circumference) was determined with a standard tape (Omrom, Los Angeles, CA, USA), located between the last rib upper and the border of the iliac crest and. Body height (cm) was determined using a height measurement scale (Omrom, Los Angeles, CA, USA). Body weight was determined while the subjects were unclothed (Omrom, Los Angeles, CA, USA). The patients attended fasting for 8 h, without smoking or prior alcohol consumption, in order to realize impedance. BMI was obtained with the next formula: weight in kilograms divided by height in squared meters. Total fat mass was obtained by impedance with an accuracy of 5 g (EFG BIA 101 Anniversary, Akern, Firenze, Italy) [10]. An alternating electric current of 0.8 mA at 50 kHz was produced by a calibrated signal generator (EFG, Akern, Firenze, Italy) The equation of this device was used: (0.756 × Height^2^/Resistance) + (0.110 × Body mass) + (0.107 × Reactance) – 5.463. Systolic and diastolic blood pressures were measured with a sphygmomanometer (Omrom, Los Angeles, CA, USA), after the participants sat for 5 min during the physical exploration with three repetitions per patient. The mean of these three determinations was used.

The FLI was calculated according to the formula published by Bedogni et al. [7]: FLI = EXP(0.953 × LN(triglyceride) + 0.139 × BMI + 0.718 × LN(GGT) + 0.053 × waist − 15.745) / (1 + EXP(0.953 × LN(triglyceride) + 0.139 × BMI + 0.718 × LN(GGT) + 0.053 × waist − 15.745)) × 100.

### 2.5. Statistical Analysis

Sample size was determined to detect differences over 5 UI/L on liver enzymes levels with 90% power and 5% significance (*n* = 600). All subjects had FLI > 60 units. These patients were analyzed in all groups, and in other two different groups: Group I ((ALT) < 43 UI/L) and group II ((ALT) ≥ 43 UI/L) (The results were shown as mean (standard deviation). The distribution of parameters was analyzed with Kolmogorov–Smirnov test. Quantitative variables with normal distribution were evaluated with a two-tailed, paired Student’s *t*-test. Non-parametric variables were evaluated with the W-Wilcoxon test. Qualitative variables were evaluated with the chi-square test (Yates’s correction and Fisher’s test were used, too). A *p* value under 0.05 was considered statistically significant.

### 2.6. Ethical Approval

All procedures were in accordance with the ethical standards of the institutional research committee (HVUVA committee 7/2020) and with the 1964 Helsinki Declaration and its later amendments. Written informed consent was obtained from all individual participants included in the study.

## 3. Results

Table 2 shows the baseline characteristics in all group and females/males. BMI, weight fat mas and waist circumferences were higher in males than females.

In the total group (*n* = 606), subjects (555 group I with ALT < 43 UI/L and 51 group II with ALT ≥ 43 UI/L) treated with pMR diet, baseline data of nutritional intake with 3-day written food report, showed a calorie intake of 1686.8 ± 89.2 kcal/day. The macronutrient distribution showed a high amount of fats (Table 3). The same caloric intake without significant differences was found in group I and II, with a similar predominance of fats in the intake (Table 2). During the dietary treatment, these participants reached the dietary targets of pMR diet, 1023.1 ± 88.2 calories, with an increase in caloric intake from carbohydrates and a decrease in fat. These same results were obtained in patients in groups I and II, without differences between the two groups (Table 2). Finally, physical activity remained without changes throughout the study in both groups.

Table 4 shows anthropometric data and blood pressure. In the whole group, pMR, BMI, weight, fat mass, waist circumference, and blood pressure improved in a significant way. The decrease at 3 months was similar in both groups (deltas group I vs. deltas group II) (BMI: −2.3 ± 0.4 kg/m^2^ vs. −2.0 ± 0.2 kg/m^2^: *p* = 0.25), weight (−8.6 ± 1.2 kg vs. −9.2 ± 1.3 kg: *p* = 0.36), fat mass (−6.9 ± 0.3 kg vs. −7.2 ± 0.2 kg: *p* = 0.54), systolic blood pressure (−10.0 ± 2.1 mmHg vs. −9.2 ± 1.3 mmHg: *p* = 0.35), and diastolic blood pressure (−4.3 ± 2.1 mmHg vs. −5.8 ± 1.2 mmHg: *p* = 0.24).

Table 5 reports the changes in biochemical parameters. There was a significant improvement in the total group of the following parameters: glucose, total cholesterol, LDL-cholesterol, triglycerides, fasting insulin, and HOMA IR. The improvement at 3 months was similar in both groups (deltas group I vs. deltas group II): glucose (−7.6 ± 0.9 mg/dL vs. −7.1 ± 0.7 mg/dL: *p* = 0.31), total cholesterol (−15.4 ± 2.8 mg/dL vs. −19.8 ± 3.9 mg/dL: *p*= 0.32), LDL-cholesterol (−8.8 ± 2.4 mg/dL vs. −13.1 ± 3.2 mg/dL: *p* = 0.39), triglycerides (−12.9 ± 1.8 mg/dL vs. −19.0 ± 3.9 mg/dL: *p* = 0.21), insulin (−5.2 ± 1.8 units vs. −7.7 ± 1.9 mg/dL: *p* = 0.29), and HOMA-IR (−1.8 0.2 units vs. −2.1 ± 0.4 units: *p* = 0.21). Basal levels of triglycerides, fasting insulin, and HOMA-IR were higher in group II than group I.

Table 6 shows the differences in biochemical liver parameters and FLI index. There was a significant decrease in the total group of the following parameters: ALT, AST, GGT, and FLI index. A significant increase in AST/ALT ratio was detected, too. The improvement at 3 months was higher in group II (deltas group I vs. deltas group II); ALT (−4.2 ± 0.9 UI/L vs. −32.1 ± 5.7 UI/L: *p* = 0.01), AST (−4.8 ± 1.8 UI/L vs. −14.1 ± 1.9 UI/L: *p* = 0.02), GGT (−4.8 ± 1.4 UI/L vs. −37.1 ± 4.2 UI/L: *p*= 0.01), and AST/ALT ratio (−0.04 ± 0.002 units vs. −0.19 ± 0.04 units: *p* = 0.01) than patients in group I. The improvement of FLI-index at 3 months was similar in both groups (deltas group I vs. deltas group II); FLI index (−10.8 ± 1.2 units vs. −9.1 ± 1.4 units: *p* = 0.23).

## 4. Discussion

In this design, we reported that a pMR diet produced a significant improvement in adiposity parameters, blood pressure, lipid profile, fasting glucose and fasting insulin levels, and biochemical liver parameters in subjects with obesity and FLI ≥ 60 units. The amount of improvement of ALT, AST, GGT, and ratio ALT/AST was higher in subjects with basal ALT > 43 UI/L.

We showed that a modest weight improvement for three months was associated with a transaminase’s decrease in these patients. In fact, based on our data, a 5–10% reduction in body weight could be pointed as an initial therapeutic target in patients with metabolic associated fatty liver disease (MAFLD), as it is recommended in the literature [1]. This weight improvement was related with the improvement of total cholesterol, LDL-cholesterol, HOMA-IR, and insulin. These results show that achieving and maintaining a 5–10% weight decrease will improve not only liver transaminases, but also several other components of the metabolic syndrome, for example, HOMA-IR, and it is related with the pathogenesis of MAFLD [11]. The explanation of the connection between insulin resistance and hepatic steatosis remains unknown. In patients with obesity, the first abnormality may genetically produce insulin resistance, with a secondary rise of serum triglyceride levels, due to the enhancement of the peripheral lipolysis. This increase in the hepatic supply of fatty acids and insulin may raise the triglyceride deposition in the liver [12], and this fatty acid deposition increases substrates for oxidative stress. Therefore, especially in the presence of insulin resistance, in which the flux of free fatty acids (FFAs) from adipose is not suppressed by insulin, the elevated rate of lipogenesis may be a significant source of stored triacylglycerol in the liver. However, the better dietary restrictions are still unclear [13,14,15]; thus, an optimal recommendation for these patients is still lacking and exploring new strategies, such as pMR diets, is necessary.

Very few designs of the effects of pMR diet on MAFLD have been realized [5,6]. In a group of 26 subjects with non-alcoholic steatohepatitis, Deibert et al. [5] reported that a meal replacement diet with a soy yogurt honey preparation for 18 weeks had similar effects on body weight and liver fat by magnetic resonance spectroscopy, compared to a comprehensive lifestyle intervention. In this study, the dietary intervention consisted of 1200 to 1800 cal per day, with 50% of the calories provided from carbohydrates, 25–30% provided from fat, and 15–20% provided from protein. The lower caloric restriction of this intervention, compared to ours, may explain the non-superiority of pMR diet over a conventional diet. Moreover, the lipid profile improved in both groups of our design, but the decrease in triglycerides was only significant in pMR group. The weight reduction in this study was inferior to ours, reaching only 5% of the initial weight. In a short-term intervention study of 2 weeks, Baltry et al. [6] reported the effectiveness of a pMR diet in severely obese subjects prior to bariatric surgery. MAFLD histology assessments post-diet showed no significant difference between pMR diet and a very low energy diet, achieving similar weight loss, reduction in inflammatory markers, and liver steatosis. In this study, the energy intake in the pMR group was lower than our present study (750 cal per day vs. 1000 cal per day), and the macronutrient distribution showed a hyperproteic diet (38% of the calories provided from carbohydrates, 25% provided from fat, and 37% provided from protein), another difference with our study. The weight reduction in this study was inferior to ours, reaching only 2.5% of the initial weight. All these differences in dietary intervention can explain the data obtained.

In a study of 12 weeks of intervention [16], Behari et al. reported that the pMR diet induced huge weight loss, which was related to the improvement of hepatic steatosis in 14 MAFLD patients. This intervention recommended approximately 1100 cal per day, including five serving of the Optifast^®®^ product (Nestle, Geneve, Switzerland), supplemented with two cups of non-starchy vegetables per day. In this study, the weight loss achieved was 12% of the initial body weight, with an important effect on microbiota, too. This previous dietary intervention [16] was very similar to the intervention of a low-calorie diet. In this topic area, there is another study [17] with a low-calorie diet for 60 weeks in obese patients to evaluate the improvement of liver enzymes. In this study, with a diet of 800 calories per day and a high protein intake of macronutrients (45% protein, 38% carbohydrate, and 17% fat), a significant long-term improvement of liver enzymes was demonstrated, secondary to a weight loss of 10%.

Although the previously reviewed studies were heterogeneous in their design, the intervention time, caloric restriction, and distribution of macronutrients, as well as the populations studied, all of them had the objective of evaluating the effect of hypocaloric diets with greater or lesser replacement of some intake on hepatic parameters. Without a doubt, the effect has been beneficial and has been shown the pMR diet as a safe dietary strategy. Another interesting approach in our study is the use of liver enzymes and the FLI index to monitor patients. Both parameters are accessible and economical for an approach in the real clinical practice of patients with MAFLD [18]; therefore, we consider it of interest. In fact, they are much cheaper than ultrasound scanning, magnetic resonance, or computed tomography scanning of the liver. They are less invasive than the current liver biopsy, too. Finally, FLI index is a useful parameter [3]. This index has been shown to be useful in monitoring liver function during dietary interventions. Cueto-Galan et al. [19] showed a decrease in this index after a dietary intervention consisting of a Mediterranean diet. Despite being an estimation measure, it has been validated in different studies and can be considered a simple tool in routine clinical practice [20,21].

A limitation of our design was the inability to study the stage of liver injury, as would be possible in histological evaluations. However, liver biopsy was not available in our population, in which the participants were asymptomatic and ethical problems could be reached. Secondly, other limitations were that biochemical variables (AST, ALT, GGT, and bilirubin) could not be associated with liver histological changes. Thirdly, the lack of a control group without diet intervention could be a bias. Finally, the presence of males and females in the sample, taking into account the potential difference in transaminase levels between both sexes [22], can be a source of confusion. However, we found no differences in the initial analysis between both groups. Further studies are needed to evaluate the effect of pMR on liver histology and liver image techniques, as well as to evaluate the effect of this interventions in patients with obesity and other comorbidities, such as diabetes mellitus and cardiovascular events.

## 5. Conclusions

In conclusion, we reported that the pMR diet is an effective method for losing weight and improving metabolic data in patients with obesity and high FLI, with both normal and elevated baseline ALT levels. The decrease in liver enzymes was greater in patients with basal ALT > 43 UI/L.

## Figures and Tables

**Table 1:** Distribution of calories and macronutrients in the partial meal replacement diet, total diet plus formula (column 1), and only formula (column 2).

	Theorical Oral Diet + Formula	Only Normocaloric Hyperproteic Formula (200 mL per Brick)
Energy (kcal)	1035	200
Proteins (g (%TCV))	64.4 (25%)	15.4 (31%)
Fats (g (%TCV))	19.1 (17%)	5.2 (23%)
Carbohydrates (g (%TCV))	151.6 (59%)	21 (42%)
Dietary Fiber (g)	15.9	4.2

Normocaloric hyperproteic formula is VEGESTART^®®^,Vegenat, Badajoz, Spain; (%TCV: % total caloric value).

**Table 2:** Anthropometric parameters and blood pressure at baseline time (mean ± standard deviation) at basal time.

Parameters	All group (*n* = 666)	Females (*n* = 442)	Males (*n* = 164)	*p* Value between Male and Females
BMI	40.3 ± 4.3	39.3 ± 2.1	40.6 ± 2.1	*p* = 0.03
Weight (kg)	103.2 ± 4.5	100.1 ± 4.0	107.9 ± 4.1	*p* = 0.02
Fat mass (kg)	47.1 ± 2.1	47.9 ± 3.8	42.1 ± 3.0	*p* = 0.03
WC (cm)	120.9 ± 2.1	118.8 ± 3.2	121.9 ± 3.0	*p* = 0.01
SBP (mmHg)	136.3 ± 2.1	136.0 ± 3.1	136.9 ± 3.0	*p* = 0.21
DBP (mmHg)	82.4 ± 4.0	82.2 ± 3.1	83.1 ± 3.2	*p* = 0.33
Glucose (mg/dL)	109.2 ± 2.1	108.8 ± 1.9	110.1 ± 1.8	*p* = 0.44
Total cholesterol (mg/dL)	198.1 ± 7.7	197.1 ± 4.2	199.6 ± 4.2	*p* = 0.39
LDL-cholesterol (mg/dL)	119.1 ± 3.1	118.9 ± 2.8	121.9 ± 4.1	*p* = 0.42
HDL-cholesterol (mg/dL)	50.1 ± 3.0	50.4 ± 3.1	49.9 ± 2.0	*p* = 0.31
Triglycerides (mg/dL)	139.4 ± 4.0	137.1 ± 3.9	141.1 ± 4.2	*p* = 0.32
Insulin (mUI/L)	20.1 ± 1.8	19.9 ± 1.9	22.5 ± 3.9	*p* = 0.28
HOMA-IR	5.4 ± 0.3	5.3 ± 0.5	5.5 ± 0.9	*p* = 0.42
ALT (U/L)	25.3 ± 2.1	21. 9 ± 1.1	27.3 ± 4.1	*p* = 0.31
AST (U/L)	22.6 ± 2.0	19.9 ± 2.8	23.8 ± 4.1	*p* = 0.49
GGT (U/L)	36.4 ± 2.1	32.9 ± 2.0	37.6 ± 4.9	*p* = 0.34
Total bilirubin (mg/dL)	0.6 ± 0.1	0.6 ± 0.2	0.7 ± 0.4	*p* = 0.31
AST to ALT ratio	0.95 ± 0.09	0.98 ± 0.08	0.95 ± 0.09	*p* = 0.27
FLI	92.8 ± 3.8	92.6 ± 3.1	93.8 ± 2.0	*p* = 0.41

Table 2 shows anthropometric variables, biochemical values and blood pressure in total group, females, and males. Last column: Statistical differences between gender. BMI: body mass index; DBP, diastolic blood pressure; SBP, systolic blood pressure; WC, waist circumference. LDL, Low density lipoprotein; HDL, High density lipoprotein; HOMA-IR, Homeostasis model assessment insulin resistance; ALT, Alanine amino transferase; AST, aspartate aminotransferase activity; GGT, Gama glutamine transferase; FLI, Fatty Liver Index.

**Table 3:** Average daily intakes and physical activity at baseline time and after 3 months of intervention (mean ± standard deviation).

Daily Intakes	Group I ALT < 43 UI/L (*n* = 555)			Group II ALT ≥ 43 (*n* = 51)			
	Basal	3 Months	Effect Size	Basal	3 Months	Effect Size	*p* Values
Calorie intake (kcal/day)	1699.2 ± 78.1	1021.1 ± 61.1 *	609.8±48.9	1695.2 ± 92.1	1028.1 ± 30.4 *	611.2 ± 41.1	*p* = 0.34 *p* = 0.02 *p* = 0.01
Carbohydrate intake (g/day) (PTC %)	168.1 ± 23.0 (40.3%)	130.3 ± 31.9$ (63.2%)	37.1±13.1	169.9 ± 23.1 (39.9%)	131.9 ± 30.0$ (63.0%)	38.1 ± 9.0	*p* = 0.43 *p* = 0.02 *p* = 0.01
Fat intake (g/day) (PTC %)	57.2 ± 11.3 (36.5%)	27.8 ± 8.1 # (22.7%)	29.2 ± 4.3	57.8 ± 11.0 (36.7%)	27.5 ± 8.5# (22.8%)	22.2 ± 5.1	*p* = 0.52 *p* = 0.03 *p* = 0.04
Protein intake (g/day) (PTC %)	72.9 ± 12.3 (23.2%)	56.2 ± 8.9 & (23.3%)	16.1 ± 7.3	72.0 ± 13.1 (23.4%)	56.8 ± 11.9& (23.4%)	15.9 ± 6.1	*p* = 0.33 *p* = 0.02 *p* = 0.01
Fiber intake (g/day)	15.9 ± 5.0	16.9 ± 4.1	1.1 ± 0.3	15.6 ± 5.1	16.0 ± 4.1	0.9 ± 0.2	*p* = 0.21 *p* = 0.57 *p* = 0.18
Physical activity (min/week)	129.8 ± 7.9	130.8 ± 10.9	1.1 ± 0.9	128.7 ± 7.1	131.1 ± 10.4	2.1 ± 1.9	*p* = 0.29 *p* = 0.41 *p* = 0.39

PTC: Percentage of total calorie (* Daily Calorie intake; $ Daily Carbohydrate intake; # Daily fat intake; & Daily protein intake). Statistical differences *p* < 0.05, in each group with basal group. Statistical differences in the last column: first *p* indicates effect size between groups; second *p* indicates time effect for group I; third *p* indicated time effect for group II.

**Table 4:** Anthropometric parameters and blood pressure in different groups (mean ± standard deviation) at baseline time and after 3 months of intervention.

Parameteres	Group I ALT < 43 UI/L (*n* = 555)			Grop II ALT ≥ 43 (*n* = 51)			
	Basal	3 Months	Effect Size	Basal	3 Months	Effect Size	*p* Values
BMI	40.3 ± 2.2	37.0 ± 2.0 *	2.3 ± 1.2	39.8 ± 2.0	35.8 ± 1.9 *	3.0 ± 1.3	*p* = 0.34 *p* = 0.03 *p* = 0.02
Weight (kg)	102.9 ± 4.1	94.3 ± 2.3 $	8.9 ± 1.1	106.4 ± 4.0	96.1 ± 2.2 $	9.9 ± 1.1	*p* = 0.51 *p* = 0.03 *p* = 0.03
Fat mass (kg)	47.5 ± 3.0	40.7 ± 2.2 #	6.5 ± 1.1	43.4 ± 3.1	36.2 ± 2.0 #	6.7 ± 1.0	*p* = 0.23 *p* = 0.02 *p* = 0.02
WC (cm)	120.8 ± 3.1	112.9 ± 2.2 &	7.8 ± 1.1	121.3 ± 3.1	110.1 ± 2.2 &	9.8 ± 2.1	*p* = 0.41 *p* = 0.02 *p* = 0.03
SBP (mmHg)	136.2 ± 3.0	126.4 ± 4.0 **	10.2 ± 1.1	137.1 ± 3.0	128.3 ± 2.1 **	9.3 ± 1.0	*p* = 0.43 *p* = 0.01 *p* = 0.02
DBP (mmHg)	82.1 ± 3.1	78.4 ± 1.3 ***	3.1 ± 1.1	85.3 ± 3.1	78.9 ± 2.1 ***	5.1 ± 2.3	*p* = 0.29 *p* = 0.03 *p* = 0.03

BMI: body mass index; DBP, diastolic blood pressure; SBP, systolic blood pressure; WC, waist circumference; (* BMI, $ Weight, # fat mass, & WC, ** SBP, *** DBP). Statistical differences *p* < 0.05, in each group with basal data. Statistical differences in the last column: first *p* indicates effect size between groups; second p indicates time effect for group I; third p indicates time effect for group II.

**Table 5:** Biochemical parameters in different groups (mean ± standard deviation) at basal time and after 3 months of intervention.

Biochemical Parameters	Group I ALT < 43 UI/L (*n* = 555)			Group II ALT ≥ 43 (*n* = 51)			
	Basal	3 Months	Effect Size	Basal	3 Months	Effect Size	*p* Values
Glucose (mg/dL)	108.8 ± 1.9	101.2 ± 2.2+	7.2 ± 1.2	113.8 ± 1.9	106.3 ± 2.2 +	7.2 ± 0.9	*p* = 0.47 *p* = 0.04 *p* = 0.01
Total cholesterol (mg/dL)	197.9 ± 4.5	182.2 ± 3.1 *	15.2 ± 1.1	203.6 ± 4.1	183.8 ± 2.1*	19.9 ± 1.3	*p* = 0.59 *p* = 0.02 *p* = 0.02
LDL-cholesterol (mg/dL)	119.9 ± 2.0	111.1 ± 1.2 $	8.1 ± 0.4	123.5 ± 4.1	110.8 ± 3.1 $	13.1 ± 1.0	*p* = 0.47 *p* = 0.03 *p* = 0.02
HDL-cholesterol (mg/dL)	51.0 ± 3.0	49.9 ± 2.1	2.1 ± 0.1	46.9 ± 3.0	44.9 ± 2.1	3.9 ± 0.9	*p* = 0.39 *p* = 0.60 *p* = 0.33
Triglycerides (mg/dL)	137.5 ± 3.2	125.8 ± 2.1 #	12.1 ± 2.0	167.1 ± 10.2 ++	136.8 ± 9.9 #	11.8 ± 1.9 #	*p* = 0.42 *p* = 0.02 *p* = 0.01
Insulin (mUI/L)	19.7 ± 1.9	14.5 ± 1.3 &	5.2 ± 1.3	25.5 ± 1.9++	17.7 ± 2.1 &	9.5 ± 3.3	*p* = 0.38 *p* = 0.03 *p* = 0.01
HOMA-IR	5.3 ± 0.3	3.6 ± 0.4 **	1.6 ± 0.2	7.2 ± 0.4++	5.0 ± 0.3 **	2.2 ± 0.5	*p* = 0.34 *p* = 0.02 *p* = 0.01

HOMA-IR (Homeostasis model assessment). Statistical differences *P*<0.05, in each genotype group (+ glucose, * Total cholesterol, $ LDL-cholesterol, # Triglycerides, & insulin, ** HOMA IR). Statistical differences *p* < 0.05, in each group with basal data ++ Statistical differences between group I and II in basal levels of insulin *p* = 0.02, HOMA-IR *p* = 0.03 and triglycerides *p* = 0.01. Statistical differences in the last column: first *p* indicates effect size between groups; second *p* indicates time effect for group I; third *p* indicates time effect for group II. LDL, Low density lipoprotein; HDL, High density lipoprotein;

**Table 6:** Liver parameters in different groups (mean ± standard deviation) at basal time and after 3 months of intervention.

	Basal	3 Months	Effect Size	Basal	3 Months	Effect Size	*p* Values
ALT (U/L)	21.9 ± 1.1	19.7 ± 1.2 +	2.7 ± 0.2	70.3 ± 4.1 **	38.4 ± 7.2 +, **	32.7 ± 1.9 ##	*p* = 0.03 *p* = 0.03 *p* = 0.01
AST (U/L)	19.9 ± 0.8	16.1 ± 1.1 *	3.1 ± 1.0	41.9 ± 4.9* *	27.1 ± 3.1*,**	23.1 ± 1.8 ##	*p* = 0.39 *p* = 0.04 *p* = 0.01
GGT (U/L)	32.9 ± 2.0	28.1 ± 1.7 $	4.1 ± 0.7	87.6 ± 4.1**	50.9 ± 3.9$,**	37.1 ± 2.9 ##	*p* = 0.42 *p* = 0.02 *p* = 0.01
Total Billirubin (mg/dL)	0.6 ± 0.2	0.7 ± 0.1	0.1 ± 0.1	0.7 ± 0.2	0.6 ± 0.1	0.1 ± 0.1	*p* = 0.39 *p* = 0.60 *p* = 0.33
AST to ALT ratio	0.99 ± 0.08	1.03 ± 0.06 #	0.02 ± 0.01	0.61 ± 0.08	0.80 ± 0.06 #	0.18 ± 0.06 ##	*p* = 0.43 *p* = 0.03 *p* = 0.02
FLI	92.6 ± 3.1	82.8 ± 2.9 &	9.8 ± 0.2	95.8 ± 2.1	83.6 ± 3.9 &	12.8 ± 3.9	*p* = 0.55 *p* = 0.03 *p* = 0.01

ALT: alanine aminotransferase; AST: aspartate aminotransferase; GGT: gamma-glutamiltransferase; FLI: Fatty liver index (+, ALT; *, AST; $, GGT; #, AST/ALT; &, FLI). Statistical differences *p* < 0.05, in each group with basal data. ** Statistical differences between group I and II in basal levels of ALT *p* = 0.02, AST *p* = 0.03, and GGT *p* = 0.01. Statistical differences in the last column: first *p* indicates effect size between groups; second *p* indicates time effect for group I; third *p* indicates time effect for group II.

## Data Availability

The data that support the findings of this study are available from the corresponding author upon reasonable request.

## References

[1] Bilson J., Sethi J.K., Byrne C.D. (2021). Non-alcoholic fatty liver disease: a multi-system disease influenced by ageing and sex, and affected by adipose tissue and intestinal function. Proc. Nutr. Soc..

[2] Berná G., Romero-Gomez M. (2020). The role of nutrition in non-alcoholic fatty liver disease: Pathophysiology and management. Liver Int..

[3] Gutiérrez-Fisac J.L., Guallar-Castillon P., León-Muñoz L.M., Graciani A., Banegas J.R., Rodríguez-Artalejo F. (2011). Prevalence of general and abdominal obesity in the adult population of Spain, 2008–2010: The ENRICA study. Obes. Rev..

[4] Heymsfield S.B., Mierlo C.A.J.V., Van Der Knaap H.C.M., Heo M., Frier H.I. (2003). Weight management using a meal replacement strategy: Meta and pooling analysis from six studies. Int. J. Obes. Relat. Metab. Disord..

[5] Deibert P., Lazaro A., Schaffner D., Berg A., Koenig D., Kreisel W., Baumstark M.W., Steinmann D., Buechert M., Lange T. (2019). Comprehensive lifestyle intervention vs. soy protein-based meal regimen in non-alcoholic steatohepatitis. World J. Gastroenterol..

[6] Baldry E.L., Aithal G.P., Kaye P., Idris I.R., Bennett A., Leeder P.C., Macdonald I.A. (2017). Effects of short-term energy restriction on liver lipid content and inflammatory status in severely obese adults: Results of a randomized controlled trial using 2 dietary approaches. Diabetes Obes. Metab..

[7] Borggreve S.E., Hillege H.L., Wolffenbuttel B.H.R., de Jong P.E., Bakker S.J.L., van der Steege G., van Tol A., Dullaart R.P.F., PREVEND Study Group (2005). The effect of cholesteryl ester transfer protein -629C->A promoter polymorphism on high-density lipoprotein cholesterol is dependent on serum triglycerides. J. Clin. Endocrinol. Metab..

[8] Friedewald W.T., Levy R.I., Fredrickson D.S. (1972). Estimation of the Concentration of Low-Density Lipoprotein Cholesterol in Plasma, Without Use of the Preparative Ultracentrifuge. Clin. Chem..

[9] Matthews D.R., Hosker J.P., Rudenski A.S., Naylor B.A., Treacher D.F., Turner R.C. (1985). Homeostasis model assessment: Insulin resistance and beta-cell function from fasting plasma glucose and insulin concentrations in man. Diabetologia.

[10] Lukaski H.C., Johnson P.E., Bolonchuk W.W., Lykken G.I. (1985). Assessment of fat-free mass using bioelectrical impedance measurements of the human body. Am. J. Clin. Nutr..

[11] Meneghel P., Pinto E., Russo F.P. (2022). Physiopathology of nonalcoholic fatty liver disease: From diet to nutrigenomics. Curr. Opin. Clin. Nutr. Metab. Care.

[12] Sanyal A.J., Campbell Sargent C., Mirshashi F., Rizzo W.B., Contos M.J., Sterling R.K., Luketic V.A., Shiffman M.L., Clore J.N. (2001). Nonalcoholic steatohepatitis: Association of insulin resistance and mitocohondrial abnormalities. Gastroenterology.

[13] Aller R., Sigüenza R., Pina M., Laserna C., Antolín B., Burgueño B., Durà M., Izaola O., Primo D., de Luis D.A. (2020). Insulin resistance is related with liver fibrosis in type 2 diabetic patients with non-alcoholic fatty liver disease proven biopsy and Mediterranean diet pattern as a protective factor. Endocrine.

[14] Rico D., Martin-Diana A.B., Lasa A., Aguirre L., Milton-Laskibar I., De Luis D.A., Miranda J. (2019). Effect of Wakame and Carob Pod Snacks on Non-Alcoholic Fatty Liver Disease. Nutrients.

[15] Aller R., Izaola O., Gómez S., Tafur C., González G., Berroa E., Mora N., González J.M., De Luis D.A. (2015). Effect of silymarin plus vitamin E in patients with non-alcoholic fatty liver disease. A randomized clinical pilot study. Eur. Rev. Med. Pharmacol. Sci..

[16] Behari J., Graham L., Wang R., Schirda C., Borhani A.A., Methé B.A., Li K., Morris A., Luu H.N., Palmieri S. (2020). Dynamics of hepatic steatosis resolution and changes in gut microbiome with weight loss in nonalcoholic fatty liver disease. Obes. Sci. Pr..

[17] Gasteyger C., Larsen T.M., Vercruysse F., Astrup A. (2008). Effect of a dietary-induced weight loss on liver enzymes in obese subjects. Am. J. Clin. Nutr..

[18] Sebastiani G., Patel K., Ratziu V., Feld J.J., A Neuschwander-Tetri B., Pinzani M., Petta S., Berzigotti A., Metrakos P., Shoukry N. (2022). Current considerations for clinical management and care of non-alcoholic fatty liver disease: Insights from the 1st International Workshop of the Canadian NASH Network (CanNASH). Can. Liver J..

[19] Cueto-Galán R., Barón F.J., Valdivielso P., Pintó X., Corbella E., Gómez-Gracia E., Wärnberg J. (2017). Changes in fatty liver index after consuming a Mediterranean diet: 6-Year follow-up of the PREDIMED-Malaga trial. Medicina Clinica.

[20] Cuthbertson D.J., Weickert M.O., Lythgoe D., Sprung V.S., Dobson R., Shoajee-Moradie F., Umpleby M., Pfeiffer A.F., Thomas E.L., Bell J.D. (2014). External validation of the fatty liver index and lipid accumulation product indices, using 1H-magnetic resonance spectroscopy, to identify hepatic steatosis in healthy controls and obese, insulin-resistant individuals. Eur. J. Endocrinol..

[21] Balkau B., Lange C., Fezeu L., Tichet J., de Lauzon-Guillain B., Czernichow S., Fumeron F., Froguel P., Vaxillaire M., Cauchi S. (2008). Predicting diabetes: Clinical, biological, and genetic approaches: Data from the Epidemiological Study on the Insulin Resistance Syndrome (DESIR). Diabetes Care..

[22] Mera J.R., Dickson B., Feldman M. (2007). Influence of Gender on the Ratio of Serum Aspartate Aminotransferase (AST) to Alanine Aminotransferase (ALT) in Patients With and Without Hyperbilirubinemia. Dig. Dis. Sci..

